# Policy recommendations to ensure that research software is openly accessible and reusable

**DOI:** 10.1371/journal.pbio.3002204

**Published:** 2023-07-21

**Authors:** Erin C. McKiernan, Lorena Barba, Philip E. Bourne, Caitlin Carter, Zach Chandler, Sayeed Choudhury, Stephen Jacobs, Daniel S. Katz, Stefanie Lieggi, Beth Plale, Greg Tananbaum

**Affiliations:** 1 Open Research Funders Group, Washington, DC, United States of America; 2 Departamento de Física, Facultad de Ciencias, Universidad Nacional Autónoma de México, Mexico City, Mexico; 3 Mechanical and Aerospace Engineering, George Washington University, Washington, DC, United States of America; 4 School of Data Science, University of Virginia, Charlottesville, Virginia, United States of America; 5 Higher Education Leadership Initiative for Open Scholarship (HELIOS), Washington, DC, United States of America; 6 Center for Open and Reproducible Science, Stanford Data Science, Stanford University, Stanford, California, United States of America; 7 Open Source Programs Office, Carnegie Mellon University, Pittsburgh, Pennsylvania, United States of America; 8 Open@RIT, Rochester Institute of Technology, Rochester, New York, United States of America; 9 National Center for Supercomputing Applications, Computer Science, Electrical & Computer Engineering, and iSchool, University of Illinois Urbana-Champaign, Champaign, Illinois, United States of America; 10 Open Source Program Office, University of California, Santa Cruz, Santa Cruz, California, United States of America; 11 Data to Insight Center, Indiana University, Bloomington, Indiana, United States of America

## Abstract

Research data is optimized when it can be freely accessed and reused. This Perspective provides recommendations for policymakers to maximize research equity, transparency, and reproducibility, ensuring that research software is openly accessible and reusable.

## Teaser

Research data is optimized when it can be freely accessed and reused. To maximize research equity, transparency, and reproducibility, policymakers should take concrete steps to ensure that research software is openly accessible and reusable.

In August 2022, the White House Office of Science and Technology Policy (OSTP) issued a memorandum on “Ensuring Free, Immediate, and Equitable Access to Federally Funded Research” [1], representing a transformational step toward making all United States federally funded research both immediately accessible and fully reusable. The OSTP’s memo will be especially impactful in the realm of data sharing, as it asks federal agencies to develop plans to require data underlying published studies to be shared immediately upon publication and explore strategies for sharing all data, even if not tied to a published study. This policy advance takes place within the larger context of international efforts to enable open data sharing at scale, including the national open science plans in Ireland [2], Colombia [3], Spain [4], and France [5]. As contributors to the Higher Education Leadership Initiative for Open Scholarship and the Open Research Funders Group, we believe this moment in time represents an unparalleled opportunity to elevate research software as a core component of the scientific endeavor and to take specific steps to ensure its open and equitable availability.

The OSTP guidance and its accompanying press release [6] outline the importance of applying an equity lens to US federal policy, stating, “Financial means and privileged access must never be the prerequisite to realizing the benefits of federally funded research that the American public deserves” and that “A federal public access policy consistent with our values of equal opportunity must allow for broad and expeditious sharing of federally funded research—and must allow all Americans to benefit from the returns on our research and development investments without delay.” In particular, the OSTP guidance emphasizes the critical role of data sharing to ensure transparency, validation, reproducibility, and integrity of US federally funded research. However, truly meeting these goals will not just require sharing data, but also sharing the research software needed to open and reanalyze data. As explored by the FAIR for Research Software working group (jointly convened by the Research Data Alliance, FORCE11, and the Research Software Alliance), we are using the term “research software” to encompass “source code files, algorithms, scripts, computational workflows and executables that were created during the research process or for a research purpose” [7]. We acknowledge that this definition may continue to evolve through ongoing community discussions, as well as with advances in AI, simulations, algorithms, etc.

Research data, especially in certain specialized fields, is often collected and stored in proprietary file formats that require software with costly licenses to open and analyze. If data are shared under such conditions, without accompanying code, algorithms, and software to allow others to open and analyze the files, this will represent a financial barrier to access that will delay or prohibit reuse of data, especially for underserved populations, citizen scientists, and early career researchers with less means. It might also present technical barriers that could limit the machine readability and reusability of data by assistive devices. All of this would run counter to the guiding principles and overall goals of the 2022 OTSP guidance to make data Findable, Accessible, Interoperable, and Reusable (FAIR). Most importantly, to accurately be able to replicate and reproduce results and build on shared data, we must not only have access to the data themselves, but also understand exactly how they were used and analyzed.

Requiring research software to be shared as part of an integral data sharing policy is not without precedent within the US federal government. The CHIPS and Science Act of 2022 [8] includes language on the need for federal grantees to describe how they will archive and preserve research software as part of their data management plans. The National Institutes for Health’s Strategic Plan for Data Science [9] emphasizes that “Extracting understanding from large-scale or complex biomedical research data requires algorithms, software, models, statistics, visualization tools, and other advanced approaches such as machine learning, deep learning, and artificial intelligence.” In response to the OSTP memo, the Department of Transportation has stated their intention to add “Source Code and Software, among the categories of accessible Research Outputs” to their forthcoming updated public access plan [10]. NASA’s Science Mission Directorate has also released an update to its comprehensive Scientific Information Policy (SPD-41a) [11], which applies to a significant portion of NASA’s research expenditures, and which requires that research data and software are shared openly at the time of publication or by the end of the funding award. There is now an opportunity to expand US federal policies in similar ways and align their research software sharing aspects across agencies.

To do this, we recommend:

1. As part of their updated policy plans submitted in response to the 2022 OSTP memo, US federal agencies should, at a minimum, articulate a pathway for developing guidance on research software sharing, and, at a maximum, incorporate research software sharing requirements as a necessary extension of any data sharing policy and a critical strategy to make data truly FAIR (as these principles have been adapted to apply to research software [12]).
2. As part of sharing requirements, federal agencies should specify that research software should be deposited in trusted, public repositories that maximize discovery, collaborative development, version control, long-term preservation, and other key elements of the National Science and Technology Council’s “Desirable Characteristics of Data Repositories for Federally Funded Research” [13], as adapted to fit the unique considerations of research software.
3. US federal agencies should encourage grantees to use non-proprietary software and file formats, whenever possible, to collect and store data. We realize that for some research areas and specialized techniques, viable non-proprietary software may not exist for data collection. However, in many cases, files can be exported and shared using non-proprietary formats or scripts can be provided to allow others to open files.
4. Consistent with the US Administration’s approach to cybersecurity [14], federal agencies should provide clear guidance on measures grantees are expected to undertake to ensure the security and integrity of research software. This guidance should encompass the design, development, dissemination, and documentation of research software. Examples include the National Institute of Standards and Technology’s secure software development framework and Linux Foundation’s open source security foundation.
5. As part of the allowable costs that grantees can request to help them meet research sharing requirements, US federal agencies should include reasonable costs associated with developing and maintaining research software needed to maximize data accessibility and reusability for as long as it is practical. Federal agencies should ensure that such costs are additive to proposal budgets, rather than consuming funds that would otherwise go to the research itself.
6. US federal agencies should encourage grantees to apply licenses to their research software that facilitate replication, reuse, and extensibility, while balancing individual and institutional intellectual property considerations. Agencies can point grantees to guidance on desirable criteria for distribution terms and approved licenses from the Open Source Initiative.
7. In parallel with the actions listed above that can be immediately incorporated into new public access plans, US federal agencies should also explore long-term strategies to elevate research software to co-equal research outputs and further incentivize its maintenance and sharing to improve research reproducibility, replicability, and integrity.

While the context of these recommendations is, given the timeline of the OSTP memorandum [1], primarily focused on US federal agencies, the general principles may be adapted and adopted by a range of public and private funders, regardless of geography. In making these recommendations, we recognize that considerations for sharing research software relate to, but do not precisely overlap with, considerations for sharing research papers and data. The ways in which research software is developed, when in the project lifecycle it can be productively shared, and how (and by whom) it is maintained represent just a few of the differences. As a consequence, a uniform approach for “sharing research objects” that treats research software as fundamentally interchangeable with research papers and data will not succeed. A well-developed US federal strategy in this area will require significant consideration of the ways in which overarching principles such as FAIRness, sustainability, open infrastructure, curation good practices, and expansive licensing must be adapted to address the unique parameters of research software.
